# Psychometric analysis of the brief symptom inventory 18 (BSI-18) in a representative German sample

**DOI:** 10.1186/s12874-016-0283-3

**Published:** 2017-01-26

**Authors:** Gabriele Helga Franke, Susanne Jaeger, Heide Glaesmer, Claus Barkmann, Katja Petrowski, Elmar Braehler

**Affiliations:** 1 Department of Rehabilitation Psychology, University of Applied Sciences Magdeburg-Stendal, Osterburger Str. 25, 39576 Hansestadt Stendal, Germany; 2 Department of Medical Psychology and Medical Sociology, University of Leipzig, Leipzig, Germany; 3 Department of Child and Adolescent Psychiatry, University Medical Center Hamburg-Eppendorf, Hamburg, Germany; 4 Department of Preventive Research, German Sport University Cologne, Cologne, Germany

**Keywords:** Psychological distress, BSI-18, Reliability, Factorial structure

## Abstract

**Background:**

The BSI-18 contains the three six-item scales somatization, depression, and anxiety as well as the Global Severity Index (GSI), including all 18 items. The BSI-18 is the latest and shortest of the multidimensional versions of the Symptom-Checklist 90-R, but its psychometric properties have not been sufficiently clarified yet.

**Methods:**

Based on a representative sample of *N* = 2516 participants (aged 14–94 years), detailed psychometric analyses were carried out.

**Results:**

The internal consistency was good: Somatization α = .82, Depression α = .87, Anxiety α = .84 and GSI α = .93. Confirmatory factor analysis supported the three scales as second-order and GSI as first-order factors. The model fit based on RMSEA is good but that model fit based on CFI and TLI are too low.

**Conclusions:**

Therefore, it is a very short, reliable instrument for the assessment of psychological distress. The BSI-18 can be used to reliably assess psychological distress in the general population. However, further studies need to evaluate the usefulness of standardization in clinical samples.

## Background

Internationally, the SCL-90 [16, 21, 22] is the most used questionnaire for the assessment of psychological distress, especially in clinical practice [45]. However, it is a very long and time-consuming questionnaire, which is why two Brief Symptom Inventory versions of the Symptom Checklist SCL-90-R were developed [12, 18, 20, 21]: the Brief Symptom Inventory with 53 items (BSI; [13, 17, 19, 23]), which measures psychological distress, and its shortened version, the Brief Symptom Inventory with 18 items (BSI-18; [11, 14, 24]).

The Brief Symptom Inventory with 53 items was developed by Derogatis using a factor analysis and maintaining the scale structure with the reduced item number of the SCL-90-R (somatization, obsessive-compulsive, interpersonal sensitivity, depression, anxiety, anger-hostility, phobic anxiety paranoid ideation and psychoticism). In Germany, the BSI is mainly used for quality management in psychotherapy (e.g. [28]).

In order to reduce and prevent an overload to the patients and to ensure an easy screening-tool, the BSI-18 was developed with highest clinical relevance. The BSI-18 contains only the three six-item scales somatization (SOMA), anxiety (ANX), depression (DEPR), and the global Scale Global Severity Index (GSI). (They are documented in Table 1). Contrary to the SCL-90-R and the BSI-53, the BSI-18 scores were calculated by sum scores. The GSI therefore ranges between 0 – 72 and the three scales between 0 – 24. The application studies demonstrated that the BSI-18 is a suitable instrument for measuring psychological distress and comorbidities in patients with different mental and somatic illnesses (e.g. [1, 4, 8, 9, 10, 29, 38, 39, 46, 48]). This instrument is also used in longitudinal studies [5, 6, 37].

**Table 1:** Item- and scale statistics, reliability, and convergent validity in the total sample (*N* = 2516)

No	Item	*M* (*SD*)	Corrected item-total correlation	α Without the item	PHQ-Depression correlations	PHQ-Anxiety correlations
Scale 1: Somatization (α = .82)		*∑*1.46 (2.58)			.52**	.48**
1	Faintness or dizziness	0.19 (0.51)	.56	.80		
4	Pains in heart or chest	0.23 (0.56)	.61	.79		
7	Nausea or upset stomach	0.28 (0.63)	.49	.81		
10	Trouble getting your breath	0.22 (0.57)	.65	.78		
13	Numbness or tingling in parts of your body	0.22 (0.60)	.55	.80		
16	Feeling weak in parts of your body	0.33 (0.69)	.66	.77		
Scale 2: Depression (α = .87)		*∑*1.76 (3.23)			.72**	.71**
2	Feeling no interest in things	0.36 (0.69)	.67	.85		
5	Feeling lonely	0.42 (0.82)	.66	.85		
8	Feeling blue	0.27 (0.67)	.78	.83		
11	Feelings of worthlessness	0.26 (0.69)	.74	.83		
14	Feeling hopeless about the future	0.39 (0.83)	.73	.84		
17	Thoughts of ending your life	0.07 (0.36)	.51	*.88*		
Scale 3: Anxiety (α = .84)		*∑*1.44 (2.59)			.63**	.72**
3	Nervousness or shakiness inside	0.27 (0.62)	.66	.80		
6	Feeling tense or keyed up	0.50 (0.77)	.59	.83		
9	Suddenly scared for no reason	0.17 (0.50)	.62	.81		
12	Spells of terror or panic	0.10 (0.39)	.66	.82		
15	Feeling so restless you couldn’t sit still	0.23 (0.60)	.66	.80		
18	Feeling fearful	0.19 (0.53)	.63	.81		
Global Severity Index (α = .93)		*∑*4.66 (7.44)			.71**	.73**

** = *p* < .0001

Until now, there have only been three studies which address the applicability and psychometric properties of the German version of the BSI-18 in patients after renal transplantation [26] and in hospitalized psychosomatic patients [25, 49].

In contrast, the psychometric properties of the BSI-18 were discussed internationally in 13 publications. The reliability (Cronbachs α) ranged for SOMA between α_*min*_ = .61 [36] and α_*max*_ = .84 [50], for DEPR between α_*min*_ = .64 [36] and α_*max*_ = .92 [43], for ANX between α_*min*_ = .71 [2] and α_*max*_ = .88 [50] and for the GSI between α_*min*_ = .84 [36] and α_*max*_ = .94 [43]. The reliability was mostly above .80 and can thus be evaluated as good. The reliability for the American norm sample (*N* = 1134; α-SOMA = .74, α-DEPR = .84, α-ANGS = .79, α -GSI = .89; [14]) is to be rated as satisfactory.

The retest-reliability for *n* = 103 psychological distressed patients after 15 days without intervention was satisfactory with values between *r*_*tt*_ = .68 and *r*_*tt*_ = .82 [2]. For validity evidence based on internal structure, a strong first factor was discussed (e.g. [3]) alike to the SCL-90-R and the BSI-53 [42]. Based on an exploratory factor analysis, the original 3-scale structure could be replicated in *n* = 638 hospitalized psychosomatic patients [25]. In addition, the original scale structure was often tested by confirmatory factor analysis [25, 26, 50]. Convergent validity was shown in several studies [2, 49]. Sensitivity and specificity were first analyzed by Zabora et al. [51] using the BSI-53.

As yet, psychometric properties based on a representative sample are still not available for Germany. Therefore, the aim of this study was to (1) describe the psychological distress within the German population, to present (2) the reliability, and (3) the factorial validity.

## Methods

### Data acquisition

A representative sample of the general population in Germany was collected in November/ December 2009 by a demography-consulting company (USUMA, Berlin). A total of 258 sample points were used (210 in the western part and 48 in the eastern part of Germany). The households and members of these households were selected via random-route procedure. The sample was representative for the German population regarding age, gender, and education as proved by comparisons with the Federal Statistical Office. To begin with, 4091 addresses were selected; 22% had to be dropped as neutral (e.g. persons unknown), and 38% could not be asked (e.g., due to illness, holidays, refusal, non-availability). In the end, a total of 2520 persons could be included in the sample.

## Materials

### Sample description

The representative sample contains 2516 individuals (53.7% female) with an average of 50.5 years of age (SD = 18.6, Range = 14 – 94 years). A total of seven nearly equidistant age groups were set up: ages 14–24 (10.7%), 25–34 (11.6%), 35–44 (16.3%), 45–54 (17.3%), 55–64 (16.5%), 65–74 (17.5%), and 75–94 (10%). In the sample, 52% were married, 23.4% single, 11.3% divorced, and 13.2% separated. Employment: 37.9% had a full-time job, and 9.2% had a part-time job. The remainder of the sample was unemployed (8.1%), retired (33.9%), housewife/ house-husband (5.3%), and 9.7% had not yet completed their education. Educational background: 44.1% had a lower education, 36.3% an upper education, and 6.8% an advanced education; 6.6% were university students, 4.1% were still attending school, and 2% had not graduated.

### Psychological assessments

Demographic information, the BSI-18, and further psychological assessments were collected in the survey. To investigate validity evidence based on external criteria, the 4-item version of the Patient Health Questionnaire was used to screen for depression and anxiety (PHQ-4; [32–34]). All the questions apply to the two preceding weeks and are to be rated by using “0 = not at all”, “1 = several days”, “2 = more than half the days” and “3 = nearly every day”. For statistical calculations, the answer category “0” was to be opposed to the other three categories.

### Statistics

The analyses were carried out using PASW and AMOS. First, a Missing Data Analysis led to the exclusion of four participants because they showed more than the tolerated amount of missing data (tolerated < 1 items of each scale, < 3 items in total). At last, a total of 0.09% of the answers were missing and not assigned randomly (Little MCAR-Test: *Chi-Quadrat* = 550.971, *df* = 333, *p* < .0001). Therefore they were replaced by using Multiple Imputation (MCMC in LISREL 8.15; [35]).

Descriptive statistics, reliability as well as discriminant and convergent correlations were estimated. Construct validity was tested by using the confirmatory factor analysis (CFA).

Using AMOS [31], the respective fit of the two-factor and the three-factor model was tested using CFAs. Due to the lack of multivariate normality in the data tested with the Marida-test in AMOS, the Asymptotically Distribution Free-estimator (ADF) was used for model testing [7]. According to Schermelleh-Engel, Moosbrugger, and Müller [47], a good (acceptable) model fit is a given with SB χ^2^/df index below 2.0 (below 3.0), Comparative Fit Index (CFI) as well as Tucker-Lewis-Index (TLI) above .95 (above .90), Standardized Root Mean Square Residual (SRMR) below .05 (below .10), and Root Mean Square Error of Approximation (RMSEA) below .05 (below .08).

## Results

### Psychological distress, reliability, and convergent validity of the scales

The mean values of the 18 items and sum scores of the three scales and the GSI had a left-skewed distribution (see Table 1), Table 2 reported gender- and age differences. Internal consistency was α = .82 for SOM, α = .87 for DEPR, α = .84 for ANX and α = .93 for the GSI. The corrected discriminatory power was only below .50 for item no. 7 (nausea or upset stomach). Furthermore, the elimination of item no. 17 (thoughts of ending your life) would increase the reliability of the scale DEPR. The Depression scale of the PHQ correlated the highest with DEPR (*r* = .72), followed by substantial correlations with GSI (*r* = .71), ANX (*r* = .63), and the lowest with SOM (*r* = .52). The Anxiety scale of the PHQ correlated quite equal with GSI (*r* = .73), ANX (*r* = .72), and DEPR (*r* = .71), but the lowest with SOM (*r* = .48).

### Factorial validity

The confirmatory factor analysis was used to prove the theoretical and empirical structure of the BSI-18. Due to the lack of multivariate normality in the data tested with the Marida-test in AMOS, the Asymptotically Distribution Free-estimator (ADF) was used for model testing. The three factor model (SOMA, DEPR and ANX) resulted in *χ* ^*2*^ = 355, *df* = 132, *p* < 0.001; *RMSEA* = .030 [.02 - .03]; *TLI* = .48; *CFI* = .55 (see Table 1).

Two different models were tested by using the ADF-method and the software AMOS; model modifications were not tolerated: the theoretical one-factor-model (*χ* ^*2*^ = 526,696, df = 136, *p* < 0.001; RMSEA = .034 [.031 - .037]; TLI = .12; CFI = .22, Standardized RMR = .332) and the three-factor-model with SOMA, DEPR and ANX (*χ* ^*2*^ = 355,143, df = 132, *p* < 0.001; RMSEA = .026 [.023 - .029]; TLI = .483; CFI = .554, Standardized RMR = .138). The theoretical three-scale structure with the GSI as main factor was the model tested last (*χ* ^*2*^ = 355,143, df = 132, *p* < 0.001; RMSEA = .026 [.023 - .029]; TLI = .483; CFI = .554, Standardized RMR = .138).

**Table 2:** Gender- and Age differences in the BSI-18 scales and the GSI

Variable	SOMA	DEPR	ANX	GSI
Gender	*F* = 1.7 *p* < 0.20	*F* = 7.2 *p* < 0.007	*F* = 8.5 *p* < 0.004	*F* = 6.9 *p* < 0.009
		*η* ^*2*^ = 0.003	*η* ^*2*^ = 0.003	*η* ^*2*^ = 0.003
Men (*n* = 1163)	1.36 (2.4)	1.56 (3.1)	1.26 (2.4)	4.19 (6.9)
Women (*n* = 1353)	1.54 (2.7)	1.92 (3.3)	1.59 (2.7)	5.07 (7.8)
Age	*F* = 36.0 *p* < 0.0001	*F* = 5.2 *p* < 0.0001	*F* = 2.1 *p* < 0.05	*F* = 11.0 *p* < 0.0001
	*η* ^*2*^ = 0.079	*η* ^*2*^ = 0.012	*η* ^*2*^ = 0.005	*η* ^*2*^ = 0.026
I: 14–24 years (*n* = 270)	0.85 (2.2) I < V VI VII	1.57 (3.7) I < VII	1.40 (3.0)	3.83 (8.3) I < VII
II: 25–34 years (*n* = 293)	0.70 (1.8) II < V VI VII	1.27 (2.5) II < VII	1.09 (2.1)	3.05 (5.7) II < VI VII
III: 35–44 years (*n* = 410)	0.79 (1.7) III < V VI VII	1.36 (2.7) III < VII	1.35 (2.3)	3.50 (5.8) III < VII
IV: 45–54 years (*n* = 436)	1.27 (2.4) IV < VI VII	2.0 (3.5)	1.59 (2.6)	4.85 (7.6) IV < VII
V: 55–64 years (*n* = 415)	1.59 (2.6) V > I II III V < VII	1.89 (3.2)	1.54 (2.8)	5.02 (7.6) V < VII
VI: 65–74 years (*n* = 440)	2.07 (2.8) VI > I II III IV VI < VII	1.77 (3.1)	1.35 (2.4)	5.19 (7.4) VI > II VI < VII
VII: 75–94 years (*n* = 252)	3.17 (3.5) VII > I II III IV V VI	2.57 (3.8) VII > I II III	1.76 (3.0)	7.50 (9.0) VII > I II III IV V VI
Gender x Age	*F* = 1.2 *p* < 0.30	*F* = 0.7 *p* < 0.68	*F* = 0.9 *p* < 0.50	*F* = 0.8 *p* < 0.56

*Comments. Mean values and standard deviations for scale sums and the GSI are presented for gender, age and their interaction.* Effect sizes (*η* ^*2*^) or post hoc tests are presented if the result is significant

## Discussion

Up to now, the BSI-18 has not been used widely in Germany. The psychometric properties and benefits of the instrument were investigated in three samples [25, 26, 49]. For the present representative sample, the questions concerning reliability and model fit could be answered.

The reliability (Cronbach’s α) of the BSI-18 (α-SOMA = .82, α-DEPR = .87, α-ANX = .84, α-GSI = .93) was good to very good and ranged higher than in the US standardization. The reliability of the American norm sample (*N* = 1134; α-SOMA = .74, α-DEPR = .84, α-ANGS = .79, α -GSI = .89; [14]) had to be rated as satisfactory. Therefore, it can be concluded that the internal consistency of the scales can be affected by a sufficient sample procedure [41]. The internal consistency of the scale Depression could be increased by eliminating item 17 (thoughts of ending your life). This result is similar to that of other samples, but due to the clinical relevance the item should be retained.

Using the two-item scales Depression and Anxiety of the PHQ-4 [30], to analyze convergent validity, the results were quite similar to the results by Spitzer et al. [49] using a longer PHQ-version. On the one hand, corresponding BSI-18- and PHQ-subscales demonstrated highest correlations; on the other hand, the Anxiety scale of PHQ-4 correlated similarly with BSI-18-Anxiety and BSI-18-Depression. Non-corresponding scales like the BSI-18-SOMA showed lower correlations. The results by Spitzer et al. [49] and our own results were found in non-clinical samples. Regarding clinical data [25, 26], it could be concluded that the BSI-18 is more suitable to psychologically distressed than non-distressed populations.

Congruent with international [27, 40, 42, 50] and German clinical studies [25, 26] the three scales of the BSI-18 showed the best model fits by reproducing the scale structure using the confirmatory factor analysis. Nevertheless, the boundaries for a good model fit according to Schermelleh-Engel, Moosbrugger, and Müller [47] could not be reached. The model fit based on RMSEA is good but that model fit based on CFI and TLI are too low.

The remarkable strength of the present sample is its good age distribution due to representative sampling: − young (*n* = 270, aged 14 – 24), elderly (*n* = 440, aged 65 – 74), and old age (*n* = 252, aged 75 – 94). Besides the strength of a large sample size as a limitation, it is not possible to draw general conclusions based on the data from a representative sample since a large sample size could easily lead to significant effects. Since the sample was representative for the normal population, the results are not offhandedly applicable to highly distressed samples [15]. In turn, the BSI-18 should be applied to different clinical samples to further replicate or reprobate the factorial structure.

In future research it would be productive to test the stability of the distress construct (test-retest reliability) and to explore connections to other distress questionnaires (convergent validity) or external ratings (criterion validity) [44]. A design with repeated measurements would allow for the comparison of factor structures across time and the determination of possible cohort effects.

The available version of the used software to measure the factor analysis with categorical indicators was applied. This should be seen as a limitation of this study and advice for future research.

## Conclusion

The BSI-18 is a very short, reliable instrument for the assessment of psychological distress. The factorial structure of the instrument is very good when using confirmatory factor analyses as well as the psychometric criteria. Therefore, it is an instrument that can be used to reliably assess psychological distress in clinical samples as well as in the general population. In addition, it can be used in psychotherapy research as well as in quality assurance for psychotherapeutic long-term effects. Taking into account the good internal consistency reliability estimates and the encouraging convergent validity estimates, this preliminary validation is a good step forward in validation studies which are iterative in nature.

## Abbreviations

ADF: Asymptotically distribution free-estimator
ANX: Anxiety
BSI-18: Brief Symptom Inventory (Short Version)
BSI-53: Brief Symptom Inventory
CFA: Confirmatory factor analysis
CFI: Comparative fit index
DEPR: Depression
GSI: Global Severity Index
LISREL: Linear structural relations, statistical software package
MCMC: Markov chain Monte Carlo
PASW: Predictive analytics software
PHQ-4: Patient Health Questionnaire-4
RMSEA: Root mean square error of approximation
SCL-90-R: Symptom Checklist-90-revised
SOMA: Somatization
SRMR: Standardized root mean square residual
TLI: Tucker-Lewis-index
USUMA: USUMA Sozial- und Marktforschungsinstitut
